# Addressing Underinsurance for Children With Special Health Care Needs

**DOI:** 10.1001/jamanetworkopen.2023.48857

**Published:** 2023-12-01

**Authors:** Jennifer K. Saper, Matthew M. Davis, Kristin Kan

**Affiliations:** Mary Ann and J. Milburn Smith Child Health Outcomes, Research and Evaluation Center, Stanley Manne Children’s Research Institute, Ann and Robert H. Lurie Children’s Hospital of Chicago, Chicago, Illinois; Division of Advanced General Pediatrics and Primary Care, Department of Pediatrics, Ann and Robert H. Lurie Children’s Hospital of Chicago and Northwestern University Feinberg School of Medicine, Chicago, Illinois; Nemours Children’s Health, Wilmington, Delaware; Mary Ann and J. Milburn Smith Child Health Outcomes, Research and Evaluation Center, Stanley Manne Children’s Research Institute, Ann and Robert H. Lurie Children’s Hospital of Chicago, Chicago, Illinois; Division of Advanced General Pediatrics and Primary Care, Department of Pediatrics, Ann and Robert H. Lurie Children’s Hospital of Chicago and Northwestern University Feinberg School of Medicine, Chicago, Illinois

Underinsurance among children and adolescents is a worsening problem in the United States. In 2010, an estimated 19% of US children were underinsured, representing 14 million children. However, a recent study estimated the underinsured proportion of children at 33% or approximately 24 million in 2019.^1,2^

Children are considered underinsured when, despite having health insurance coverage, they or their families experience prohibitive out-of-pocket costs, narrow networks of physicians, other clinicians, and hospitals that limit access, or challenges with enrollment and churn. These challenges can affect children with public and commercial insurance coverage. For example, the current unwinding of public insurance coverage from continuous enrollment provisions during the COVID-19 pandemic has led to more than 1.5 million children being procedurally disenrolled from Medicaid, and they must then re-enroll in their states’ programs to reestablish coverage. Churning off and on insurance contributes to underinsurance because families may forgo care while awaiting re-enrollment. Rising out-of-pocket costs have been reported among children with commercial plans, especially high-deductible health plans (HDHPs). These HDHPs can lead families to have their children go without prescription medications that have high out-of-pocket costs.

Validova et al^3^ examine the pervasive and critical problem of underinsurance as it affects families with children with special health care needs (CSHCN). To characterize underinsurance among families with CSHCN with varying needs, the authors stratified the risk of underinsurance for specific subgroups using a previously recommended framework.^4^ They distinguished CSHCN status for those with physical and mental conditions from those with only medication needs. They also identified 2 additional subgroups based on functional limitations that might occur with children’s physical and mental health conditions. This disaggregation into 5 mutually exclusive categories provides an illuminating approach for separating the patients’ type and severity of illness and the differential burden they experience from underinsurance. Validova et al^3^ found that 32% of children overall were underinsured from 2016 to 2021, and among CSHCN, who account for 18% of children overall, children with physical or mental conditions and functional limitations had the highest rates of underinsurance (41%). Children with physical health conditions (37%) and mental health conditions (38%) had higher rates of underinsurance than children with ongoing medication needs alone (32%), who were about as likely to experience underinsurance as children without special health care needs (31%). Among families with CSHCN overall, the proportion of underinsurance was highest (37%) among middle-income families (ie, annual income of 200%-399% FPL), compared with CSHCN from lower- and higher-income households.

Although the study features the challenges of underinsurance among families with CSHCN, we do not have other key information that might further characterize the burden experienced by families. The data source for the study (National Survey of Children’s Health) limits our ability to quantify the financial costs of underinsurance; families might have experienced underinsurance of $100 or $10 000, and these differing magnitudes may influence their health care-seeking behaviors for their children. We also cannot contextualize whether the underinsurance stemmed from the lack of coverage of services, lack of physicians, other clinicians, and hospitals in the plan’s network, or noncontinuous enrollment in insurance—each of which requires distinct policy solutions. Other published research to date has predominantly described the problem of underinsurance through parent reports, primarily using national surveys. To prioritize policy solutions, future work should look at underinsurance through claims or electronic health record data, which may help highlight circumstances in which parents are forgoing care. Furthermore, because the study did not separate children with underinsurance from children who are uninsured, we cannot specify the degree to which some of the reported deficiencies in coverage were experienced more starkly by the uninsured than the underinsured.^5^

These concerns notwithstanding, it is essential to build on the major findings from the study by Validova et al.^3^ Thus, we propose potential policy options that can help address underinsurance among CSHCN by targeting current gaps in coverage.

First, we recommend universal sustained coverage for children, especially in the first several years of life when patterns in health and illness are often established for the life course. The 2023 published American Academy of Pediatrics (AAP) policy statement^6^ on Medicaid proposes automatic enrollment at birth for newborns, with extended periods of eligibility, including continuous enrollment from 0 to 6 years and a minimum of 2-year eligibility periods thereafter. Continuous enrollment of children helps lessen paperwork and other administrative burdens that contribute to churn on and off health insurance. Moreover, the findings of Validova et al^3^ suggest that continuous enrollment in public programs would be a promising approach to reducing underinsurance for CSCHN in the United States today.

Second, we propose combining Medicaid and Children’s Health Insurance Program (CHIP) into a single entitlement program. Currently, the CHIP program differs from the Medicaid program because it is a block grant. This means states can impose caps and cost-sharing, which largely affects individuals with incomes that are too high for Medicaid but too low for commercial insurance. These families in the middle-income range are among the highest risk of underinsurance.^3^ Combining these 2 programs into 1 entitlement program would increase the number of children eligible for a single entitlement program, without caps or cost sharing.^6^

Third, we see great value in improving medication coverage within plans for CSHCN. There are a few mechanisms through which this might be accomplished. One policy option is gap coverage targeting medications, akin to a pharmaceutical-focused Medigap plan or Medicare Part D, which would cover specific medications to help lessen the financial burden of costly drugs for CSHCN. Another option is improvement of medication coverage for children enrolled in HDHPs, because parents often forgo medication on HDHP plans, and this can lead to increased hospitalizations for children.^7^ These plans could expand coverage of specific pediatric medications, like preventive asthma medications, through expanded drug coverage lists that emphasize prevention of exacerbations of chronic conditions common among CSHCN.

Fourth, implementing adequate coverage of all mental and behavioral health diagnoses across all plans is crucial. Validova et al^3^ describe the higher odds of underinsurance among children with mental or behavioral conditions, with or without functional limitations, compared with children with medication needs only. These children may not qualify for supplemental Medicaid coverage through waivers and may have high out-of-pocket costs for medications and therapies. Insurance plans should work to ensure network adequacy by having a sufficient number of mental and behavioral health specialists who specifically provide care for children, including developmental therapies and mental health counseling.

Approximately 1 in 3 CSHCN are underinsured and at risk of forgoing necessary care, therapies, and medications, which may impact their immediate and long-term health. These children may be overlooked when describing the state of health insurance in the US because they may be falsely assumed to have adequate access to necessary health care just because they have coverage, which is especially concerning. Ensuring that CSHCN in all families have access to timely, high-quality care must be an essential cornerstone of insurance design for protecting the health of children in the US and promoting better health throughout their lives.
